# Anticardiac Antibodies in Patients with Chronic Pericardial Effusion

**DOI:** 10.1155/2016/9262741

**Published:** 2016-01-28

**Authors:** Konstantinos Karatolios, Sabine Pankuweit, Anette Richter, Volker Ruppert, Bernhard Maisch

**Affiliations:** Department of Internal Medicine-Cardiology, Philipps University of Marburg, 35043 Marburg, Germany

## Abstract

*Objectives*. Chronic pericardial effusion may be challenging in terms of diagnosis and treatment. Specific laboratory parameters predicting the frequency and severity of recurrences after initial drainage of pericardial effusion are lacking.* Materials and Methods*. Pericardial fluid (PF) and serum (SE) samples from 30 patients with chronic pericardial effusion (PE) who underwent pericardiocentesis and pericardioscopically guided pericardial biopsy were compared with SE and PF samples from 26 control patients. The levels of antimyolemmal (AMLA) and antifibrillary antibodies (AFA) in PE and SE from patients with pericardial effusion as well as PF and SE from controls were determined and compared.* Results*. AMLAs and AFAs in PF and SE were significantly higher in patients with chronic pericardial effusion than in the control group (AMLAs: *p* = 0,01 for PF and *p* = 0,004 for serum; AFAs: *p* < 0,001 for PF and *p* = 0,003 for serum). Patients with recurrence of PE within 3 months after pericardiocentesis had significantly higher levels of AMLAs in SE (*p* = 0,029) than patients without recurrence of PE.* Conclusions*. The identification of elevated anticardiac antibodies in PE and SE indicates increased immunological reactivity in chronic pericardial effusion. High titer serum levels of AMLAs also correlate with recurrence of pericardial effusion.

## 1. Introduction

Chronic, nonmalignant, and noninfectious pericardial effusions generally have a good overall prognosis. However, recent published data have shown that even with mild pericardial effusion (PE) the overall prognosis may be worse than in age- and sex-matched controls [1]. Moreover, resistance to medical treatment and recurrence of pericardial effusion are often a therapeutic challenge to the clinician [2, 3]. So far, specific laboratory parameters predicting the frequency and severity of recurrences after initial drainage of pericardial effusion are lacking. In the current investigation, we assessed the levels of anticardiac antibodies (antimyolemmal and antifibrillary antibodies) in pericardial fluid and serum as potential markers of activity in patients with chronic pericardial effusion and epimyocarditis after exclusion of a malignant, metabolic, or systemic disease and compared them with samples of patients with coronary artery disease as control acquired during standard CABG surgery.

## 2. Materials and Methods

We investigated pericardial fluid (PF) and serum (SE) samples from 30 consecutive patients with chronic pericardial effusion (PE) who underwent pericardiocentesis and pericardioscopically guided pericardial biopsy for therapeutic and/or diagnostic reasons after signed informed consent. As control, pericardial fluid from 26 patients was obtained directly after incision of the pericardium during open heart surgery for coronary artery disease together with a serum sample for each patient. None of the patients of the control group had a history of pericardial disease or signs of pericardial effusion in preoperative echocardiography. All patients with pericardial effusion received colchicine as anti-inflammatory treatment (0,5 mg bid) [4] and were followed up for early recurrences of pericardial effusion within the first 3 months after initial drainage. None of the 30 patients was lost to the follow-up period of 3 months. Diagnosis of recurrence was established by echocardiographic documentation of reaccumulation of pericardial fluid and clinical symptoms.

**Figure 1:**
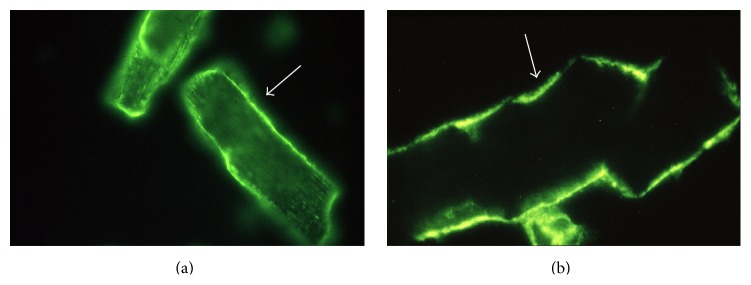
Example for the detection of antimyolemmal antibodies in pericardial fluid from a patient with chronic pericardial effusion by indirect immunofluorescence (magnification 1 : 200 for Figure 1(a) and 1 : 400 for Figure 1(b)).

To determine the levels of antimyolemmal (AMLA) and antifibrillary antibodies (AFA) in PF and SE from patients with pericardial effusion as well as PF and SE from controls we used the indirect immunofluorescence technique with isolated rat cardiomyocytes as antigen [5] (Figure 1). SE and PF samples of patients and controls (1 : 50 dilution in phosphate buffered saline) were incubated for 20 minutes with the acetone-fixed isolated cardiomyocytes. Immunoglobulin isotypes of anticardiac antibodies specific for cardiac antigens were determined with antihuman polyvalent immunoglobulin (IgP), IgG, IgM, and IgA FITC-conjugated F(ab)2-fragments from the goat (Medac). A positive result indicated the presence of specific anticardiac antibodies in the serum or pericardial effusion in a given patient. Levels of AMLAs or AFAs were expressed as 0 = negative (titer 1 ≤ 10), 0-1 = weakly positive (titer 1 ≤ 20), 1 = positive (titer 1 : 40), and 2 = strongly positive (titer 1 ≥ 80).

To exclude the presence of cardiotropic viral or bacterial agents in all samples, we used the polymerase chain reaction (PCR). For extraction of DNA/RNA from PF, SE, and pericardial biopsies, the QIAamp Blood Mini Kit and the QIAamp Tissue Kit (Qiagen, Hilden, Germany) were used. PCR for all samples was performed with primer pairs specific for influenza virus A/B, Parvovirus B19, cytomegalovirus, enterovirus, adenovirus, human herpes virus 6, and Epstein Barr virus as well as the bacteria* Borrelia burgdorferi* and* Chlamydia pneumoniae*. Conditions for polymerase chain reaction and primers have been described previously [6]. Positive results were confirmed by Southern Blot hybridization.

Patients with malignant pericardial effusion based on the presence of malignant cells in the PE or the presence of an invasive tumor in pericardioscopically guided pericardial biopsy specimens were excluded from the investigation. Patients who developed PE after radiation and chemotherapy or patients who had metabolic disorders, systemic autoimmune diseases, or uremia were also excluded.

Comparison of parametric variables was done by a 2-tailed Student's *t*-test. Nonparametric variables were compared using the Mann-Whitney test and categorical parameters were compared using the chi-square or Fisher's exact test as appropriate. Values below detection limit were assumed to be zero for statistical analysis. All *p* values < 0,05 were considered statistically significant. For statistical analysis, the statistical software package SigmaPlot version 11.0 was used.

## 3. Results

The demographic data and main clinical features of the patients are summarized in Table 1. The mean volume of aspirated pericardial fluid from patients with pericardial effusion was 419,83 ± 404,29 mL.

AMLAs and AFAs were detected in pericardial fluid in 28 (93%) patients with chronic pericardial effusion. All patients with chronic pericardial effusion had detectable AMLAs and AFAs in serum. Two patients with pericardial effusion had AMLAs and AFAs only in the serum. Moreover, AMLAs and AFAs were present in 25 (96%) PF and 25 (96%) SE samples of the control group. However, overall titers of AMLAs and AFAs in PF and SE were significantly higher in patients with chronic pericardial effusion than in patients of the control group (AMLAs: *p* = 0,01 for PF and *p* = 0,004 for SE; AFAs: *p* < 0,001 for PF and *p* = 0,003 for SE). Investigating the different subclasses of AMLA and AFA, patients with pericardial disease had significantly higher AFA-IgG levels in the pericardial fluid and significantly higher AFA-IgM and AFA-IgA levels in serum compared with the control group (*p* = 0,04 and *p* = 0,003, resp.) (Table 2).

**Figure 2:**
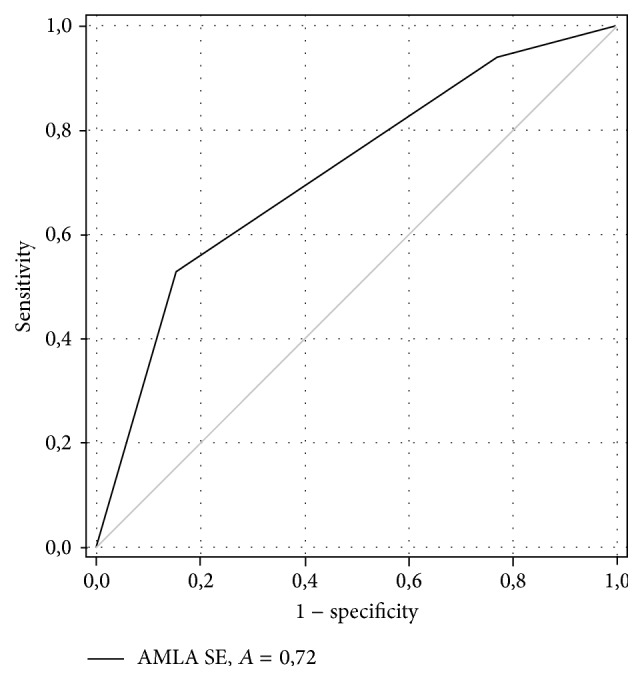
ROC curve and AUC of AMLAs in SE to discriminate patients with recurrence from patients without recurrence of pericardial effusion.

Within 3 months after pericardiocentesis with drainage of pericardial fluid, 17 patients (57%) experienced recurrence of pericardial effusion. Patients with recurrence of pericardial effusion had significantly higher levels of AMLAs in SE when compared to patients without recurrences (Table 3). However, receiver operating characteristic (ROC) analysis for AMLAs in SE demonstrated an area under the curve (AUC) of 0.72, corresponding to moderate diagnostic accuracy (Figure 2).

## 4. Discussion

In this study, we found elevated levels of AMLAs and AFAs in pericardial fluid and serum in patients with chronic pericardial effusion compared to control patients without pericardial disease indicating increased immunological reactivity in chronic pericardial effusion. In addition, patients with chronic pericardial effusion also had higher serum levels of IgM-type AFAs, indicating some sort of persistent systemic activation of the immune system.

Antimyolemmal antibodies are more frequently found in patients with perimyocarditis and dilated cardiomyopathy than in controls [7–9]. In the present study, anticardiac antibodies were found regularly in more than 90% of patients with chronic pericardial effusion. Investigations in tuberculous pericarditis suggest that AMLAs not only are diagnostic indicators of pericardial involvement but also may play a relevant role in its pathogenesis [10]. In the presence of complement, AMLAs can cause cytolysis of vital cardiomyocytes in vitro and their titers correlate with the cytolytic serum activity [7, 10, 11].

The myocardium represents the most likely source of cardiac antigens. Myocardial damage due to inflammation, ischemia, or other cardiotoxic factors may lead to release of cardiac autoantigens and subsequent production of antibodies [12, 13]. These anticardiac antibodies may then enter the pericardial space after drainage via the visceral pericardium. Therefore, the increased levels of anticardiac antibodies in the pericardial fluid may represent a marker of (epi)myocardial involvement with immunological reactivity.

In this study, patients with recurrence of pericardial effusion within 3 months after initial drainage of effusion had significantly higher levels of AMLAs in SE compared to patients without recurrences. Higher anticardiac antibody levels may result from greater antigen burden due to greater (epi)myocardial damage. A greater (epi)myocardial injury may in turn be associated with frequent recurrences. Caforio et al. reported that the presence of anti-heart antibodies in patients with recurrent pericarditis is associated with longer symptom duration and high number of relapses [14]. Moreover, pediatric patients with pericarditis and persistence of IgM-type anticardiac antibodies may face frequent recurrences of pericarditis [15].

Of note, anticardiac antibodies, albeit less elevated, were also detected in the majority of PF and SE samples of the control group. Circulating anticardiac antibodies were reported in patients with ischemic heart disease [16, 17] and the immune system may be involved in the remodelling process following myocardial infarction [17, 18]. Myocardial ischemia may cause myocardial damage and exposure of cardiac antigens with resultant antibody response. Therefore, the presence of anticardiac antibodies may indicate ongoing myocardial damage and necrosis due to myocardial ischemia.

In conclusion, identification of elevated anticardiac antibodies in pericardial effusion and serum indicates increased immunological reactivity against cardiac epitopes in chronic pericardial effusion. Higher levels of AMLAs in serum were found in patients with recurrence of pericardial effusion within 3 months after initial drainage. We interpret anticardiac antibodies in serum and pericardial effusion as markers of previous (epi)myocardial involvement in pericardial disease. Our data also indicate that high titer serum levels of AMLAs correlate with recurrence of pericardial effusion.

## Figures and Tables

**Table 1:** Demographic data and clinical characteristics of the patients (*n* = 56).

	Patients with chronic pericardial effusion	Control group
	(*n* = 30)	(*n* = 26)
Female/male	15/15	5/21
Mean age (years ± SD)	57, 29 ± 16,43	65,58 ± 9,21
LVEF (% ± SD)	52,32 ± 12,66	57,0 ± 9,5
Colchicine *n* (%)	30 (100%)	0

LV-EF: left ventricular ejection fraction; SD: standard deviation.

**Table 2:** Antimyolemmal and antifibrillary antibodies in pericardial fluid and plasma.

Autoantibody	Pericardial effusion	Control group	*p* value
	Pericardial fluid		
*AMLA (overall)*	1,57 ± 0,57	1,36 ± 0,23	**0,002**
AMLA-IgG	1,29 ± 0,25	1,18 ± 0,62	NS
AMLA-IgM	0,31 ± 0,24	0,22 ± 0,28	NS
AMLA-IgA	0,05 ± 0,15	0,07 ± 0,17	**NS**
*AFA (overall)*	1,58 ± 0,51	1,14 ± 0,32	**<0,001**
AFA-IgG	1,25 ± 0,64	0,95 ± 0,27	**0,04**
AFA-IgM	0,22 ± 0,31	0,07 ± 0,18	NS
AFA-IgA	0,03 ± 0,13	0	NS

	Serum		
*AMLA (overall)*	2,12 ± 0,34	1,81 ± 0,34	**0,004**
AMLA-IgG	1,62 ± 0,58	1,9 ± 0,25	NS
AMLA-IgM	0,63 ± 0,54	0,45 ± 0,14	NS
AMLA-IgA	0,2 ± 0,36	0,19 ± 0,25	**NS**
*AFA (overall)*	2,08 ± 0,27	1,57 ± 0,4	**<0,001**
AFA-IgG	1,65 ± 0,54	1,69 ± 0,4	NS
AFA-IgM	0,57 ± 0,58	0,14 ± 0,23	**0,003**
AFA-IgA	0,2 ± 0,39	0	**0,01**

NS: nonsignificant.

**Table 3:** Clinical features and antimyolemmal and antifibrillary antibodies in patients with and without recurrence of pericardial effusion.

	Patients with recurrence (*n* = 17)	Patients without recurrence (*n* = 13)	*p* value
Age (years)	58,71 ± 17,01	55,43 ± 16,13	NS
Female/male	11/6	4/9	NS
Pericardial effusion (mL)	366,18 ± 389,81	490 ± 427,79	NS
AMLAs in PF	1,55 ± 0,66	1,65 ± 0,43	NS
AMLAs in SE	2,24 ± 0,31	1,96 ± 0,32	0,03
AFAs in PF	1,56 ± 0,58	1,62 ± 0,42	NS
AFAs in SE	2,09 ± 0,26	1,96 ± 0,32	NS

NS: nonsignificant.
